# The Role of Imaging in Planning Treatment for Central Serous Chorioretinopathy

**DOI:** 10.3390/ph14020105

**Published:** 2021-01-29

**Authors:** Stefano Da Pozzo, Pierluigi Iacono, Alessandro Arrigo, Maurizio Battaglia Parodi

**Affiliations:** 1 TS Retina, 34170 Trieste, Italy; stefanodapozzo@yahoo.it (S.D.P.); maubp@yahoo.it (M.B.P.); 2 IRCCS—Fondazione Bietti, 00198 Rome, Italy; 3 Ophthalmology Department, San Raffaele University Hospital, 20132 Milan, Italy; alessandro.arrigo@hotmail.com

**Keywords:** central serous chorioretinopathy, fluorescein angiography, indocyanine green angiography, fundus autofluorescence, optical coherence tomography

## Abstract

Central serous chorioretinopathy (CSC) is a controversial disease both in terms of clinical classification and choice of therapeutic strategy. Choroidal layers, retinal pigment epithelium (RPE), photoreceptors, and retina are involved to varying degrees. Beyond well-known symptoms raising the clinical suspect of CSC and slit-lamp fundus examination, multimodal imaging plays a key role in assessing the extent of chorioretinal structural involvement. Subretinal fluid (SRF) originating from the choroid leaks through one or multiple RPE defects and spreads into the subretinal space. Spontaneous fluid reabsorption is quite common, but in some eyes, resolution can be obtained only after treatment. Multiple therapeutic strategies are available, and extensive research identified the most effective procedures. Imaging has carved a significant role in guiding the choice of the most appropriate strategy for each single CSC eye. Multiple biomarkers have been identified, and all of them represent a diagnostic and prognostic reference point. This review aims to provide an updated and comprehensive analysis of the current scientific knowledge about the role of imaging in planning the treatment in eyes affected by CSC.

## 1. Introduction

In many ways, central serous chorioretinopathy (CSC) still represents a somewhat mysterious disease. There is mounting scientific evidence that a combination of malfunctioning choroid (thick and hyperpermeable) and damaged retinal pigment epithelium (RPE) is the basis for this disease. In the past, imaging in CSC has been represented mainly by fluorescein angiography (FA) and indocyanine green angiography (ICGA). Newer techniques such as fundus autofluorescence (FAF), optical coherence tomography (OCT), and OCT angiography (OCT-A) have been added, enriching immensely our knowledge about mechanisms that originate CSC. Beyond a role in differential diagnosis with other potentially confounding diseases, multimodal imaging allows to plan the best treatment according to the CSC stage and the degree of choroid, photoreceptors, and RPE involvement.

**Figure 1:**
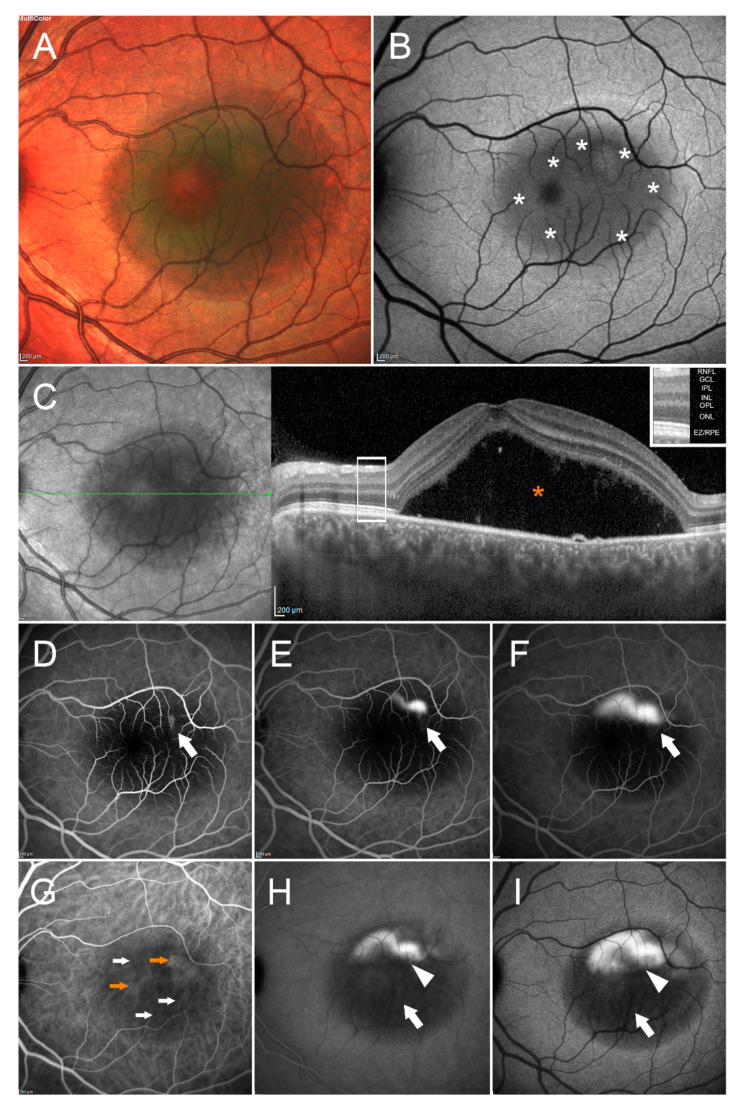
Multimodal imaging in central serous chorioretinopathy. Multicolor (**A**) image shows a mainly green round alteration involving the entire macular region. The green color may be interpreted as fluid accumulation. Blue autofluorescence (BAF) (**B**) image discloses the presence of a round hypoautofluorescent alteration (white asterisks), secondary to the masking effect made by the subretinal fluid. The presence of this latter is confirmed by structural optical coherence tomography (OCT) (**C**), showing a marked dome-shaped subretinal fluid accumulation (orange asterisk). The OCT layers legend is provided in the upper-right part of the images. FA examination discloses just a macular hypofluorescent signal on early phase (white arrow) (**D**), with the onset and enlargement of a hyperfluorescent spot in the superior macular region, detected on intermediate and late phases (white arrows) ((**E**,**F**), respectively). ICGA highlights choroidal vasculature hypercyanescent (orange arrow) and hypocyanescent (white arrow) alterations in the early phase (**G**); intermediate (**H**) and late (**I**) phases disclose a mainly hypocyanescent round signal (white arrow), with the presence of hypercyanescent superior focal leakage point (white arrowhead). The following abbreviations are used: retinal nerve fiber layer (RNFL), ganglion cell layer (GCL), inner plexiform layer (IPL), inner nuclear layer (INL), outer plexiform layer (OPL), outer nuclear layer (ONL), ellipsoid zone/retinal pigment epithelium complex (EZ/RPE).

The main clinical sign is the appearance of subretinal fluid (SRF), leaked into the subretinal space through one or multiple RPE defects. An example of multimodal imaging in an eye with acute CSC is visible in Figure 1. 

There is an increasing dye leakage on both FA and ICGA in the upper portion of the macular area, within the neurosensory detachment. The SRF masking effect can be seen as a round hypoautofluorescence on FAF, whereas both the volume and height of serous neurosensory detachment is well documented by SD-OCT. 

**Figure 2:**
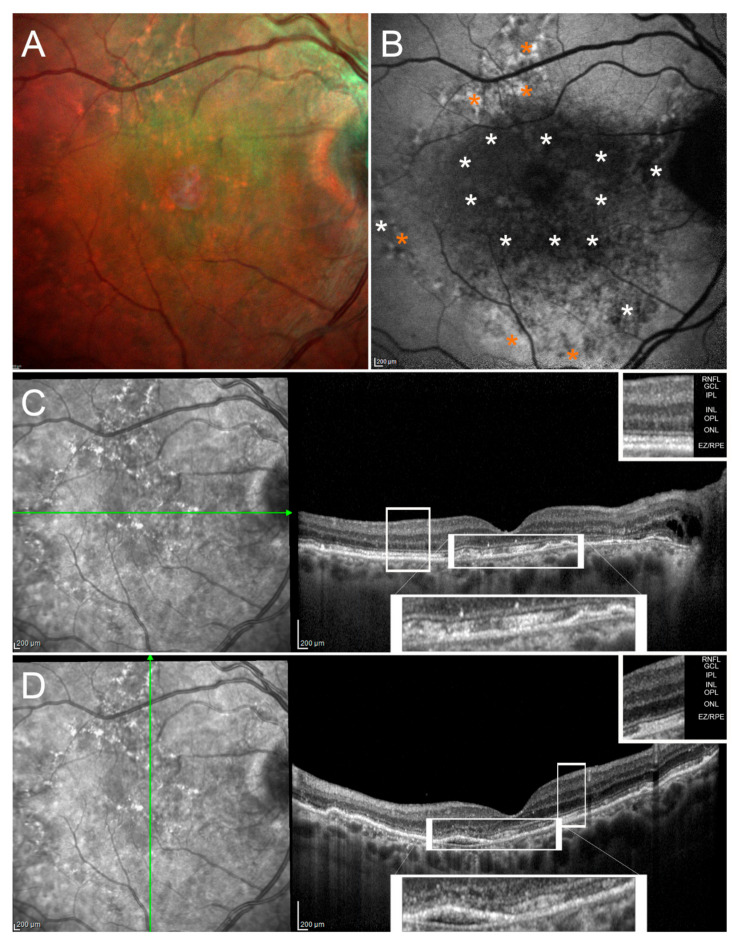
Chronic central serous chorioretinopathy. Multicolor (**A**) and BAF (**B**) images disclose sparse alterations over the entire posterior pole, characterized both by hypoautofluorescent (white asterisks) and hyperautofluorescent (orange asterisks) signals. Horizontal (**C**) and vertical (**D**) high-resolution structural OCT scans reveal the presence of diffuse alterations of the outer retinal bands, better highlighted in magnified pictures, with an irregular profile of the RPE and attenuated reflectivity signal. The OCT layers legend is provided in the upper-right part of the images. The following abbreviations are used: retinal nerve fiber layer (RNFL), ganglion cell layer (GCL), inner plexiform layer (IPL), inner nuclear layer (INL), outer plexiform layer (OPL), outer nuclear layer (ONL), ellipsoid zone/retinal pigment epithelium complex EZ/RPE).

Duration of symptoms, the detection and extent of leakage on angiography, and the degree of RPE damage are crucial information for separating acute from chronic CSC. Acute CSC is characterized by a frequent spontaneous resolution of SRF, typically within 3–4 months from the onset [1,2]. Thus, observation during this period is the commonly preferred strategy, except for those who require a faster SRF resolution and a rapid visual rescue or when photoreceptors outer segment atrophy and/or granular waste in the subretinal space are detectable in early phases of the disease. Beyond SRF resolution, an additional goal of intervention in acute CSC is to avoid a relapse. The persistence of SRF in chronic CSC is associated with potentially severe damage to photoreceptors and chronic decompensation of RPE, both followed by permanent loss of visual acuity [3,4,5]. FAF images presented in Figure 2 depict findings in an eye with chronic CSC (Figure 2).

Widespread anomalies over the entire posterior pole, characterized by a mixed pattern of both hypo- and hyper-autofluorescent areas. SD-OCT scans unveil diffuse alterations of the outer retinal bands with irregular RPE profile. 

In addition, a feared complication represented by choroidal neovascularization (CNV) may appear in the course of chronic CSC, worsening the clinical picture both in terms of diagnosis and treatment. 

## 2. Multimodal Imaging Analysis

### 2.1. The Role of Fluorescein Angiography (FA) and Indocyanine Green Angiography (ICGA)

Overall, FA anomalies are described in terms of fluorescence levels [6]. Hypofluorescence may be secondary to a blocking or a filling defect (the former caused by blood, subretinal material, or an abnormal accumulation of material in the RPE as with lipofuscin in Stargardt’s disease, the latter due to absent, decreased, or delayed normal blood flow as in retinal or choroidal vascular occlusion).

Hyperfluorescence is secondary to dye leakage, staining, pooling, or window defect. Leakage may originate from damaged vessels wall or from choroidal or retinal neovascularization, or through a dysfunctional RPE that no longer blocks fluorescence from the choroid. It is characterized by a gradual increase in size with a blurring of margins. On the contrary, staining is an increasing fluorescence throughout the angiogram whose margins remain unchanged and distinct—pooling results when fluorescein slowly fills a fluid-filled space. A window defect occurs when a layer that normally blocks fluorescence is missing (RPE is atrophic, and choroidal fluorescence is seen in FA early phases) [6]. 

ICG is a water-soluble tricarbocyanine dye that, after intravenous injection, is 98% protein-bound, so that dye diffusion through choriocapillaris small fenestrations is negligible [7]. ICG is ideal for imaging choroidal circulation due to persistency in the choroidal circulation and low permeability. Advanced systems can provide FA and ICGA images at the same time. Working at near-infrared wavelengths, ICG fluoresces better through pigment, fluid, and hemorrhage than fluorescein dye, increasing the chances of detecting abnormalities such as CNV that may be blocked by overlying blood or hyperplastic RPE on a FA [8,9]. This justifies ICGA role in imaging of occult CNV and pigment epithelial detachments (PED). CNV may appear as a plaque, a focal spot, or a combination of both. Focal spots are well-delineated fluorescent spots less than one disc diameter in size that suggest the presence of retinal angiomatous proliferations (RAP) and polypoidal choroidal vasculopathy (PCV), which are variants of CNV. 

Even if performed less frequently than in the past, FA still represents a classic technique for eyes suspects of having CSC and is helpful in identifying two key aspects about dye leakage, i.e., the leakage point and its angiographic pattern [10]. In acute CSC, a single or multiple leakage points, often within macular area, can be noted (Figure 1) [11]. An early hyperfluorescent spot, then evolving into one of the two well-described patterns, the “ink blot” or the “smoke stack” in mid-to-late phases is the usual presentation. The former is the most common (ranging from 53% to 93% of eyes) [10,11,12], the latter is typical of acute CSC in its early phases [11,12]. In eyes with chronic CSC, FA may identify a multifocal leakage or, in the alternative, a diffuse dye oozing from multiple RPE defects [12,13]. In late phases, areas of granular hyperfluorescence can be seen. Diffuse or localized RPE atrophy in eyes with resolved CSC is the cause of early hypefluorescence from window defect [14].

In eyes with CSC at different stages of evolution, SRF is commonly associated with active dye leakage. In the mid-to-late phase, the pooling of dye within the SRF may generate a circular hyperfluorescence. On the other side, an early hyperfluorescence progressively increasing in intensity may suggest the presence of a serious PED. 

**Figure 3:**
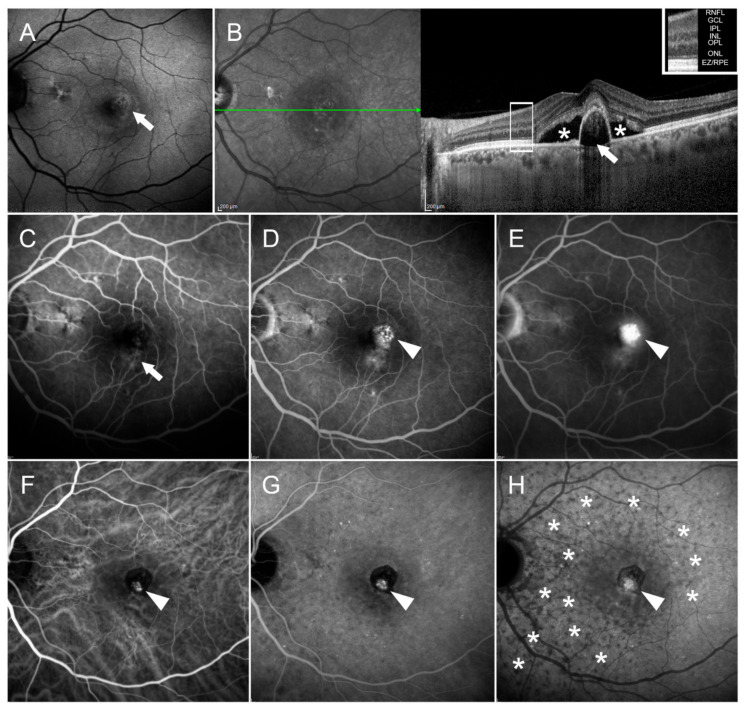
Polypoidal choroidal vasculopathy. BAF (**A**) image shows a mixed autofluorescent macular alteration (white arrow). This corresponds to subretinal fluid (white asterisk) and a PED (white arrow) on structural OCT (**B**). The OCT layers legend is provided in the upper-right part of the images. FA discloses focal RPE atrophy with window defects (white arrow) on early phase (**C**) and a hyperfluorescent lesion in the intermediate and late phases (white arrowheads) ((**D**,**E**), respectively), characterized by leakage and staining phenomena. On ICGA, in the context of sparse choroidal vascular alterations, a branching hypercyanescent network is visible on early phase (white arrowhead) (**F**), becoming more definite in the intermediate and late phases (white arrowheads) ((**G**,**H**), respectively, together with sparse hypocyanescent alterations over the entire posterior pole (white asterisks). The following abbreviations are used: retinal nerve fiber layer (RNFL), ganglion cell layer (GCL), inner plexiform layer (IPL), inner nuclear layer (INL), outer plexiform layer (OPL), outer nuclear layer (ONL), ellipsoid zone/retinal pigment epithelium complex (EZ/RPE).

ICGA still has a specific role in eyes with chronic CSC when information about choroidal vessels is needed, or a differential diagnosis with CNV or PCV is requested [15,16]. PCV is an exudative retinal disease characterized by the presence of an anomalous subretinal pigment epithelial network of vessels of choroidal origin, ending in aneurysmal dilatations [16]. Figure 3 highlights multimodal imaging findings in an eye with PCV. 

Focal RPE atrophy with window defects on the early FA phase followed by leakage and staining in intermediate and late phases can be seen, but only ICGA can visualize a branching hypercyanescent network in the early phase, becoming more definite in intermediate and late phases. FAF shows a mixed pattern of autofluorescent macular changes, corresponding to subretinal fluid and a PED on SD-OCT. 

When left undiagnosed and/or untreated, the long-term prognosis for visual function in PCV eyes is poor [17]. It is a subtype of type I or occult CNV, and ICGA is essential for accurate identification (visualization of hyperfluorescent polypoidal lesions) [18,19,20]. Distinguishing PCV is challenging due to its clinical and angiographic analogies to other pathologies (CSC, among others) [21], thus increasing the risk of the wrong therapeutic choice. 

More than 20 years ago, eyes with CSC had their choroidal vessels mapped by ICGA imaging [22,23]. A long list of anomalies (delayed filling in early phases, dilation of large vessels, and mid-phase focal hyperfluorescence, may be secondary to hyperpermeability close to leakage point) have been reported [24,25,26]. Moreover, choroidal anomalies on ICGA were observed in more than 50% of fellow asymptomatic eyes [24]. Ultra-wide-field ICGA revealed dilated choroidal vessels and hyperpermeability with congested vortex vein ampullas in >80% eyes with CSC. This suggests that outflow congestion may be a contributing factor [27]. Other authors found a significantly higher vessel density in the choroid of CSC eyes with respect to healthy control eyes [28]. In short, in CSC eyes, both choroidal structure and function are clearly altered. 

Images provided by both these angiographic techniques represent a precious reference point when clinicians opt for treating CSC eyes. Anomalies detected on both FA and ICGA are invaluable in delimiting the area to treat. Moreover, both of them offer the possibility to evaluate the treatment outcome.

In the past FA-guided focal laser photocoagulation on leakage point(s) was administered in eyes with acute CSC in order to seal RPE leak site(s) and in the attempt to accelerate SRF reabsorption, so restoring visual acuity. However, in order to avoid the onset of central or paracentral scotoma, the site of thermal treatment had to be extrafoveal. When final best-corrected visual acuity (BCVA), SRF recurrence rate, and subfoveal choroidal thickness were compared in laser-treated and untreated eyes, no statistically significant differences between groups were found [29,30,31]. These considerations may explain why focal laser treatment is no more advised as a first-line therapeutic option.

Recently a more selective procedure defined subthreshold micropulse laser treatment (STLT) was introduced. Chen et al. evaluated FA-guided STLT, and it was found to be effective in chronic CSC eyes, especially in those with a focal leakage on FA [32]. An evolution for STLT has been represented by high-density subthreshold micropulse laser (HSML), in which spots are administered in a densely packed pattern over areas with hyperfluorescent anomalies on ICGA [33,34,35]. Another available option, navigated laser photocoagulation (NLP), was proposed as a procedure for treating CSC [36,37]. Based on information from fundus photographs and FA, treatment is performed automatically by a computer on a predefined area. Complete SRF resolution has been reported in 75–94% of eyes with chronic CSC [36,37].

Van Rijssen et al. reported about another treatment option, the ICGA-guided half-dose photodynamic therapy (HD-PDT). The authors found that it generates a better response when compared to ICGA-guided HSML, regardless of the presence of focal or diffuse leakage on FA [38]. 

In the PLACE trial [35]—a large prospective, multicentre randomized trial—HSML efficacy was compared to HD-PDT in eyes with chronic CSC. Both treatments were ICGA-guided, and the authors found that SRF resolution was obtained in a significantly higher percentage of eyes after HD-PDT, both in the short-term and in the longterm. Other papers support the efficacy of HD-PDT in terms of SRF reabsorption in chronic CSC eyes [39,40,41,42]. Recently, Hayashida et al. reported that both FA- and ICGA-guided half-time PDT were effective in terms of BCVA gain, central retinal thickness (CRT), and subfoveal choroidal thickness (SCT) reduction, but ICGA-guided procedure guaranteed a significantly lower rate of SRF recurrence [43].

According to Kim et al. in eyes with acute CSC—those in which a spontaneous SRF resolution within the first 3–4 months is nonetheless quite common—ICGA-guided PDT administration is associated with a faster improvement of symptoms with respect to placebo in the short-term, but in the longterm differences become not significantly different [44]. Other authors emphasized that FA-guided HD- or half-fluence (HF)-PDT significantly decreases the SRF recurrence rate in acute CSC [45,46]. Thus, PDT should be considered as the first choice in eyes with a leakage site close to the fovea, i.e., when any laser treatment is contraindicated.

The absence of hyperfluorescent abnormalities on ICGA is a potential predictor of a post-PDT poor effect in eyes with chronic CSC with persistent serous neuroretinal detachment [47,48]. This persistence—even with associated SRF - may require a second PDT treatment but only if leakage arises from persistent—or recurrent—hyperfluorescent choroidal anomalies on ICGA, associated with focal leakage on FA.

Moreover, there is also a small subgroup of eyes with severe chronic CSC and extensive atrophic RPE changes involving the fovea on FA that should be excluded from PDT due to the risk of irreversible mild to moderate vision loss, which was reported to occur in up to 2% of eyes. At baseline, these eyes exhibit more than five optic disc diameters of diffuse atrophic RPE alterations (DARA) as visualized on mid-phase FA. This feature has been previously described as diffuse retinal pigment epitheliopathy [49].

Therefore, other than having a historical role in the diagnosis of the disease, FA and ICGA are still the guides for laser photocoagulation and PDT with verteporfin in CSC eyes. Identification of focal leakage increases the chance for a successful treatment, represented by SRF full reabsorption. The recurrence rate is a good outcome measure to evaluate treatment efficacy. 

### 2.2. The Role of Fundus Autofluorescence (FAF)

Autofluorescence (AF) is the intrinsic fluorescence emitted by a substance after being stimulated by excitation energy [50]. The clinical use of FAF in chorioretinal diseases is based on the fact that the main source of autofluorescence in the macula is lipofuscin [51]. When the RPE phagocytes photoreceptor outer segments (containing retinoids, fatty acids, and proteins), lipofuscin accumulates as an oxidative by-product. A reduction in RPE cell quantity is typically accompanied by a visible loss of autofluorescent content [52]. Thus, by analyzing these images, clinicians can indirectly postulate the RPE health levels. Hence, FAF is a non-invasive method that provides functional images of the fundus.

**Figure 4:**
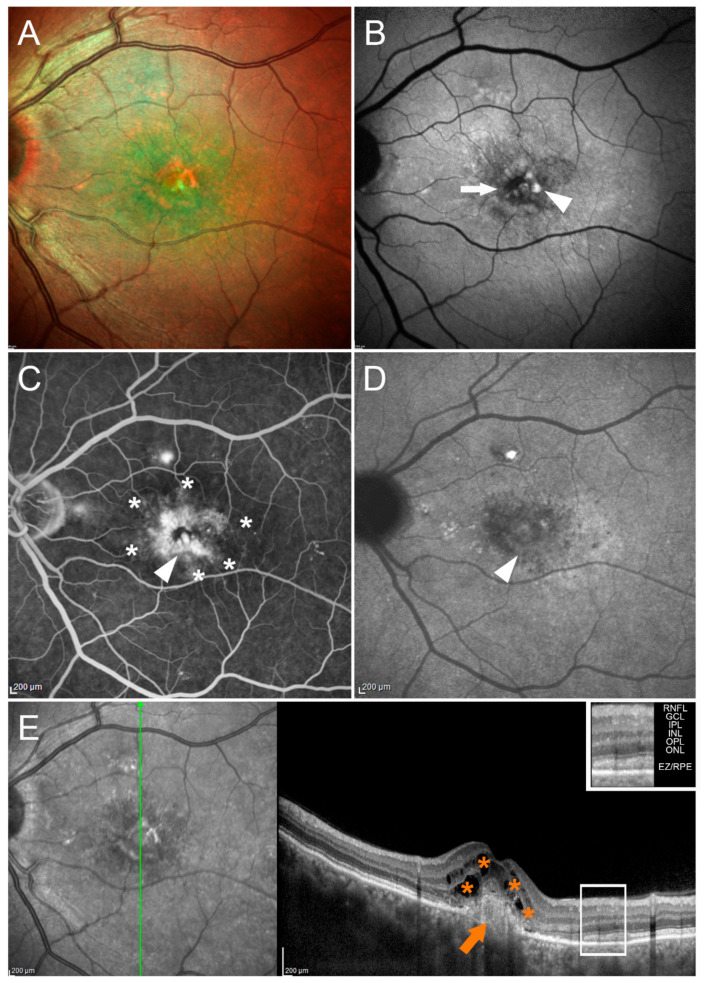
Central serous chorioretinopathy complicated by macular neovascularization. Multicolor (**A**) and BAF (**B**) images show hypoautofluorescent (white arrow) and hyperautofluorescent (white arrowhead) alterations localized in the macular region. FA (**C**) discloses a hyperfluorescent alteration (white arrowhead) with perilesional leakage (white asterisks). This corresponds to a mainly hypocyanescent alteration on ICGA (white arrowhead) (**D**). Structural OCT reveals the presence of a hyperreflective subretinal lesion (orange arrow), with smooth exudation in its context and intraretinal cysts (orange asterisks). The OCT layers legend is provided in the upper-right part of the image. The following abbreviations are used: retinal nerve fiber layer (RNFL), ganglion cell layer (GCL), inner plexiform layer (IPL), inner nuclear layer (INL), outer plexiform layer (OPL), outer nuclear layer (ONL), ellipsoid zone/retinal pigment epithelium complex (EZ/RPE) (**E**).

**Figure 5:**
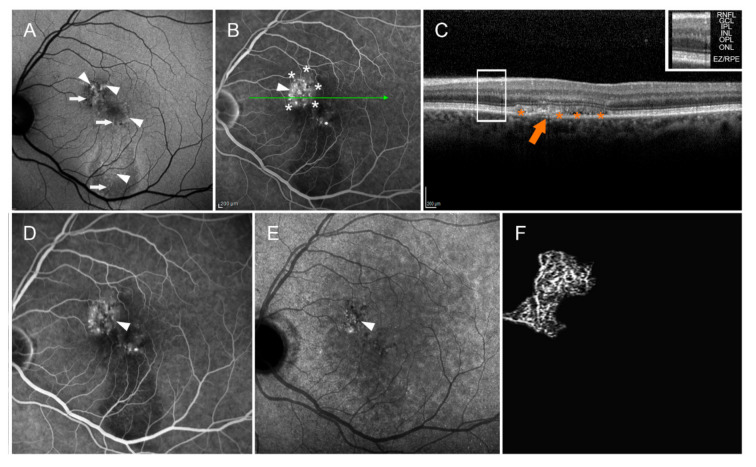
Central serous chorioretinopathy complicated by type 1 macular neovascularization. BAF (**A**) image shows the presence of macular hypoautofluorescent (white arrows) and hyperautofluorescent (white arrowheads) alterations, with a sparing of the foveal region and not homogeneous autofluorescence in the inferior part of the posterior pole, interpreted as a gravitational disposition of fluid. FA (**B**) reveals the presence of a hyperfluorescent alteration (white arrowhead) with perilesional leakage (white asterisks), which corresponds to a sub-RPE hyperreflective lesion (orange arrow) with subretinal fluid and shram (orange asterisks) (**C**). The OCT layers legend is provided in the upper-right part of the image. The hyperfluorescent lesion on FA (white arrowhead) (**D**) results mainly hypocyanescent on ICGA (white arrowhead) (**E**). OCTA well reconstructed the sub-RPE neovascular network (**F**). The following abbreviations are used: retinal nerve fiber layer (RNFL), ganglion cell layer (GCL), inner plexiform layer (IPL), inner nuclear layer (INL), outer plexiform layer (OPL), outer nuclear layer (ONL), ellipsoid zone/retinal pigment epithelium complex (EZ/RPE).

In CSC, FAF findings differ according to the stage of the disease, reflecting RPE and outer retinal changes as well as functional damage (Figure 1, Figure 2, Figure 3, Figure 4 and Figure 5). FAF may be an alternative to FA whenever the latter is contraindicated or in unusual circumstances (i.e., COVID-19 pandemic times) when it may be difficult to have time and/or available personnel to perform angiographies. Two different versions of FAF are available, short-wave FAF (SW-FAF) and near-infrared FAF (NIR-FAF). The former is generated from the RPE lipofuscin pigment (considered an indicator of RPE health), whereas the latter delineates the autofluorescence from melanin pigment of the choroid and RPE [53]. Even if NIR-FAF is used less frequently, it could represent a better predictor than SW-FAF in detecting outer retinal changes in CSC [53,54]. Coupling the two methods may provide a better interpretation of ongoing or resolved CSC episodes. When subretinal deposits are visible on FAF, foveal damage may already exist and may not be restored by administering PDT [55]. FAF imaging can help in estimating the duration of a CSC episode and the damage induced and can also support the choice for an appropriate treatment strategy [56,57].

In acute CSC, on SW-FAF there is a correspondence between hypoautofluorescence and the leakage point site on FA in a vast majority of eyes (Figure 1) [58,59]. On NIR-FAF, features are similar [60]. In chronic CSC, hypoautofluorescence on FAF continues to correspond to the leakage point on FA. A long-lasting SRF persistence is associated with increased autofluorescence levels due to persistent photoreceptor outer segment elongation, which gradually accumulates autofluorescent fluorophores as residual material [61].

Eyes with sequelae from chronic CSC may exhibit mixed FAF patterns in isolated or gravity-driven areas of RPE atrophy (Figure 2). The spectrum ranges from hyperautofluorescence (early stage with SRF) to increasing levels of hyperautofluorescence as RPE is progressively damaged by the fluid passage. The progression of FAF patterns in chronic CSC eyes is slow (the granular hypoautofluorescent pattern shifts into a confluent pattern of hypo-autofluorescence in about 24 months, on average) and then is not suitable as an outcome measure for clinical trials [62]. A recent paper identified five patterns of FAF abnormalities in a group of 126 eyes with CSC, corresponding to different levels of integrity of the ellipsoid zone and correlating with chronicity and functional damage [63]. Thereby, FAF is helpful in estimating RPE and photoreceptors outer segments health as well as in establishing a sort of structure-function relationship in both acute and chronic CSC. Various levels of damage severity (up to RPE atrophy) may be identified and a prognosis for success rate of therapy and final visual function can be made.

### 2.3. The Role of Optical Coherence Tomography (OCT) and OCT Angiography (OCT-A)

OCT is a non-invasive diagnostic technique providing in-vivo cross sectional imaging of the retina [64]. It utilizes inferometry to create an accurate cross-sectional retinal map. Spectral domain (SD) technology obtains approximately 20,000–40,000 retinal scans per second, guaranteeing a high-degree image resolution (axial resolution 3-5 microns), a higher signal-to-noise ratio, a faster acquisition time as well as the elimination of motion artifacts and a better visualization of deeper structures (i.e., the choroid) [65]. In late 2000s, SD-OCT was implemented with enhanced depth imaging (EDI) [66] and swept source (SS) technology [67]. The former takes advantage of the increased depth of field from the inverted image obtained by placing SD-OCT device close to the eye, the latter uses a wavelength-sweeping laser and a dual balanced photo detector as well as faster acquisition speeds (100,000–400,000 A-scans per seconds) at longer wavelengths of 1050-1060 nm. Nowadays, SD-OCT is the key stone imaging technique for the diagnosis and the clinical follow-up of CSC.

OCT-A is a non-invasive technique for imaging retinal and choroidal vasculature [68,69,70]. This dyeless technology uses laser light reflectance obtained from moving red blood cells to accurately depict vessels through different segmented areas of the eye. Normally an OCT scan consists of multiple individual A-scans, which when compiled into a B-scan can provide cross-sectional structural information. With OCT-A technology, the same area is repeatedly imaged and differences are analyzed between scans (over time), thus allowing one to detect areas with high flow rates (i.e., with significant changes between scans) and zones with slower, or no flow at all, which will be similar among scans.

Light is emitted through either SD-OCT (wavelength near 800 nm) or a SS-OCT (wavelength close to 1050 nm). Longer wavelengths allow a deeper tissue penetrance, but a slightly lower axial resolution. OCT-A algorithms produce an image (3 mm2 to 12 mm2) segmented by default into four zones: superficial retinal plexus, deep retinal plexus, outer retina and choriocapillaris. A potentially important OCT-A limitation is that examination is extremely motion-sensitive and requires patient collaboration, which may be difficult to obtain in the visually impaired. OCT and/or OCT-A scans in eyes with CSC may provide such a huge amount of informations about the status of the structures involved by the disease that in times of COVID-19 pandemic this may represent a sort of boost for promoting telemedicine for both diagnosis and treatment, as already done for other retinal pathologies [71].

The wealth of informations provided by OCT imaging is well summarized by Nair et al. [72]. Authors followed prospectively 100 patients with acute CSC collecting SD-OCT images. None of them received any treatment, in a sort of description of disease natural history on OCT. A PED was identified in 70% of all eyes, whereas subretinal fibrin and a rough undersurface of the neurosensory retina were noted in 61% and 64% of the eyes, respectively. A break in the PED wall and overlying fibrin were seen in 32.8% and 45% of eyes, respectively. Authors concluded that in acute CSC a poorer visual acuity is associated with a larger SRF volume (particularly a greater SRF height) and with outer nuclear layer thinning at the fovea. In resolved CSC, poorer VA was associated with a persistently thinner outer nuclear layer, shorter photoreceptor lengths, and inner/outer segment junction atrophy.

Recently, an OCT-guided (and FA-free) application of focal NLP directed on the leakage point has been tested, producing SRF resolution in the short-term [73,74]. Rajesh et al. [75] described the correspondence on SD-OCT between a hyporeflective vacuole adjacent to RPE defects and the site of active fluid leakage. This may be used as a sign of an active CSC in eyes in which FA cannot be performed or is contraindicated.

Although acute CSC often resolves spontaneously, retinal damage can still occur early in the disease course and may get worse as long as the serous retinal detachment (SRD) persists due to SRF accumulation [76]. A key information added by SD-OCT is that the SRF may not be entirely resolved. The residual subfoveal fluid can be so shallow that fundus slit-lamp biomicroscopy may miss it [77]. This residual detachment can still lead to atrophy of photoreceptor outer segments and vision loss over a period of years [78]. Moreover, OCT can detect atrophic changes into photoreceptors outer segments due to months or years of chronic foveal SRF, even in the absence of RPE abnormalities [79,80].

SD-OCT analysis have been widely used to evaluate the outcome of HSML treatment. Retinal and choroidal thickness, [81,82,83,84] resolution of SRF, [32] decrease in SRF height on OCT [84] were used as outcome measures. Overall, 36–100% of patients with chronic CSC had complete resolution of SRF after HSML treatment [32,85,86]. In the PLACE trial, HD-PDT was way better than HSML both in the short-term and in the longterm at an OCT evaluation of SRF resolution [35]. 

In the first post-PDT days, choroidal thickness can temporarily increase (up to 119% of pre-treatment thickness in 8 eyes at 2 days after treatment) on EDI-OCT [87]. At the same time, a transient increase of SRD height and worsening of visual symptoms were also reported [88,89]. These changes usually disappear at 1 month from treatment.

Some OCT-based predictive factors about PDT treatment outcome in CSC have been proposed [35,90,91,92]. In chronic CSC eyes, PDT tends to have a marginal effect and/or a high rate of SRF recurrence when one or more of the following are caught by OCT: posterior cystoid retinal degeneration (PCRD), a disruption in the ellipsoid zone and/or a shallow irregular RPE detachment. A recent study by Wu et al. conducted with OCT-A highlighted unexpected high rates of CNV after HD-PDT administered in chronic CSC eyes [93]. Patients developing post-PDT CNV had older age, larger PDT spot size, and thinner subfoveal choroidal thickness and most of the CNVs occurred within the area of previous PDT.

In order to obtain SRF resolution in eyes with disappointing response to PDT or when PDT is not available, both for acute and chronic CSC, the use of mineralocorticoid receptors (MR) antagonists may be considered. Spironolactone or eplerenone were tested. A large amount of case series and case controlled studies outlined beneficial effects of spironolactone in CSC eyes, including improved BCVA, reduced choroidal thickness, and reduced SRF [94,95,96,97,98,99,100,101,102]. Although eplerenone has more tolerable side effects with respect to spironolactone, it does not appear to be clinically superior in treating CSC [96]. Importantly, absence of CNV on OCT-A and presence of a focal leakage point (FLP) on ICGA may be positive predictive factors for complete resolution of SRF following eplerenone treatment [103]. On the other hand, patients with diffuse changes in the RPE on SD-OCT may benefit less from eplerenone treatment compared to patients who present without these abnormalities [104,105]. Based on these observations, it seems that there are three possible typologies of treatment-naive CSC patients: eyes with no CNV and presence of FLP on ICGA (SRF is likely due to an increased choroidal hyperpermeability only, thus responding almost constantly and completely to treatment); eyes with contemporary presence of both CNV and FLP, in which SRF is probably due to both an increased choroidal hyperpermeability and CNV itself, thus responding only partially to treatment; eyes characterized by presence of CNV but with no FLP, in which SRF is likely due only to the CNV itself and thus not responding to treatment. In other words, as the role of CNV increases, the response to eplerenone get worse [103]. Furthermore, OCT analysis was used to investigate choroidal thickness and central macular thickness as predictive factors of response to MRA after a relative short follow-up (3 to 6 months after treatment start). Thicker choroid at baseline was associated with a better response to treatment [104]. Several OCT parameters at baseline were identified as strong predictors for macular SRF complete resolution at 3 months, including a baseline lower number of serous PED(s) and a baseline lower number of intraretinal hyperreflective foci or dots (HRD) [106]. Cakir and associates using structural OCT and FAF reported that patients with preservation of both RPE and ellipsoid zone were those with a favorable response to oral eplerenone in terms of BCVA [105].

The VICI trial was designed to compare the safety and efficacy of eplerenone in patients with CSC [107]. This double-masked randomised placebo-controlled trial had as a primary outcome BCVA at 12 months and secondary outcomes as SRF, development of macular atrophy, subfoveal choroidal thickness, changes in low luminance visual acuity, health-related quality of life and safety. Recently the first report from this trial was released and Lotery et al. [108] stated that eplerenone was not superior to placebo for improving BCVA in people with chronic CSC after 12 months of treatment and then suggested to stop its prescription in these patients. Currently, no data about secondary outcome measures are available.

#### 2.3.1. Other Significant OCT Features in CSC Eyes

##### Pachychoroid

In all likelihood, CSC is a clinical entity belonging at least in part to the pachychoroid spectrum, a phenotype characterized by rarefaction of choriocapillaris with overlying dilated choroidal veins on EDI-OCT, as well as with progressive RPE dysfunction and increased risk of CNV [109,110,111,112]. Pachychoroid (choroidal thickness >270 μm on EDI-OCT and/or presence of pachyvessels) is secondary to enlargement of Haller’s layer and subsequent compression on inner choroid vessels [27,113,114]. Other diseases, including pachychoroid pigment epitheliopathy (PPE), pachychoroid neovasculopathy (PCN) and PCV may manifest with pachychoroid [113,114]. Nevertheless, a thicker choroid is not a mandatory diagnostic criterion for CSC. 

En face SS-OCT identified anomalies as thinning of the inner choroidal layer in the involved area (maybe due to a choriocapillaris atrophy or to compression exerted by dilated outer choroidal vessels), [109] focal or diffuse dilation of various degree, involving one or more layers [111] or areas of choroidal thickening topographically associated with pathologically dilated veins of the Haller’s layer (or “pachyvessels”), [110] abnormal hyperreflective areas involving Bruch’s membrane and choriocapillaris, corresponding to hypofluorescent areas seen on ICGA late phases [112]. As already stated above, a thicker choroid at baseline is a positive predictor for a better response to treatment with eplerenone [104]. 

##### Hyperreflective Dots (HRD)

In active chronic CSC, the presence of HRDs in inner choroid as well as hyperreflectivity of dilated vessels wall has been documented [115]. HRDs may appear also in SRF and increase in number as the disease duration increases. It is not precisely known at what HRDs do correspond, but they may be the equivalent of photoreceptor outer segments shedding, activated microglia and macrophages, or concentrated fibrin or lipid compounds. A recent study about HRDs reaffirmed that they are not a peculiar sign of CSC, since they have been described in macular dystrophies, age-related macular degeneration (AMD), retinal vein occlusion and diabetic retinopathy [116]. Authors found that in acute CSC, HRDs presence is correlated with subfoveal choroidal thickness and patients age, whereas in chronic CSC they are associated with macular and choroidal thickness as well the height of neurosensory detachment. In this latter group of eyes, HRDs could indicate an ongoing process of anatomical rearrangement of the choroid or phagocytosis of photoreceptors outer segments. Therefore, the higher the baseline HRDs number on SD-OCT, the higher the chances of recurrence after treatment [117]. Another report described that HRDs and subretinal exudates are more commonly observed in early- and late-chronic CSC than in acute CSC [118].

##### Posterior Cystoid Retinal Degeneration (PCRD)

In longlasting chronic CSC, SD-OCT may identify PCRD. It is a severe disease phenotype characterized by DARA, multiple leaking spots on FA, subretinal fibrin deposits at first presentation, [90,119,120,121,122] so creating a distinct entity within CSC, one with unfavourable visual prognosis [119]. PCRD was shown to be present in up to 35% of severe chronic CSC cases [119]. In contrast to cystoid macular edema (cystic fluid within Henle layer), PCRD may have a degenerative origin, descending from the primary choroidopathy and the RPE dysfunction, and do not stain on FA [122]. Apparently, few or no VEGF-related pathway appear involved in PCRD onset (anti-VEGF drugs are mostly ineffective in these eyes) [123]. Not rarely there is some active SRF leakage and RPE function is irreversibly damaged [124]. Treatment may help in halting some SRF leakage, but is less likely to result in a complete PCRD resolution and/or a BCVA improvement [124], since foveal damage and vision loss may be secondary to intraretinal fluid itself, as well as an associated foveal detachment [125]. In a recent report, OCT-A detected a CNV in 13 out of 29 eyes with PCRD [126].

##### Pigment Epithelial Detachment (PED)

In acute CSC a PED is identified by OCT in 53–100% of eyes [109,127,128] and not rarely colocalizes with the leakage point(s) on FA. Usually it is serous and can be found both within or outside the SRF. Its site corresponds well with areas characterized by vessels abnormalities on EDI-OCT and hyperpermeability on ICGA [109,129]. Another PED pattern identifiable in chronic CSC is defined as shallow or flat irregular PED, divided into vascularized or non-vascularized, with a potentially significant impact on treatment choice [114]. According to Song et al, low-to-flat PED(s) can be observed in all disease stages, particularly in late chronic CSC [118].

##### The Double Layer Sign (DLS)

The double-layer sign (DLS) is an OCT finding, produced by a shallow irregular PED. The upper hyperreflective band of the double layer belongs to RPE, the bottom band to the Bruch’s membrane. [130]. This finding was first reported in eyes with PCV and later in eyes with AMD and high myopia. It can be found in eyes with CSC, and usually corresponds to thinning of inner choroid and pachyvessels [109,131,132]. Risk of developing secondary CNV increases when a DLS is identifiable and some authors postulated that CSC eyes with DLS may host inactive CNV [133,134]. The space within the DLS could be hypo- or hyperreflective. DLS with internal hyporeflectivity are usually avascular, whereas an internal hyperreflectivity may host early type 1 CNV, even if it is not true in all cases [135]. Differentiating such vascularized from non-vascularized flat PED becomes difficult on conventional imaging. OCT-A has a sensitivity of 86–100% and specificity of 96–100% compared with FA in detecting CNV in eyes with chronic CSC and irregular PED [136,137]. With the advent of OCT-A, the detection of CNV in such eyes has increased significantly (up to 35%) compared with previous imaging modalities. The verified presence of an active CNV may warrant a different therapeutic approach.

##### Presence of Subretinal Fibrin

Using SD-OCT, the presence of SRF can be both assessed and quantified, which is generally considered useful for estimating the episode duration and for determining the subsequent treatment strategy [118]. OCT can reveal fibrin clots within SRF originating from fibrinogen leaked through RPE defect [138]. Abnormalities (subretinal exudation, retinal dipping, photoreceptor layes defects) were noted by the authors in close proximity to RPE defects in all 123 eyes included in the study. Subretinal fibrin can be observed as both well-defined and ill-defined hyperreflective structures [76]. A severe but unfrequent variant of chronic CSC is accompanied by bullous retinal detachment. It is generated by multiple RPE leaks and not rarely subretinal fibrin accumulates in large amount. In some cases it is accompanied by complete disruption of the PED edges, with consequent RPE avulsion (rip or tear) [139]. Another OCT feature, the retinal dragging with fibrin, has been reported, mainly in eyes with acute CSC at its early stages [118].

Liang et al. recently reported that in CSC eyes with subretinal fibrin HD-PDT with verteporfin is safe and effective, with no statistically significant difference for SRF and fibrin resolution with respect to eyes without subretinal fibrin at baseline [140].

##### CSC Complicated by CNV

In chronic CSC, OCT-A may support ICGA in the detection of CNV. Figure 4 summarizes how valuable SD-OCT can be in identifying CNV during the course of CSC.

FA shows disclose a hyperfluorescent alteration with surrounding leakage that on ICGA corresponds mainly to a hypocyanescent anomaly. SD-OCT reveals a hyperreflective subretinal lesion corresponding to CNV, with minimal exudation and intraretinal cysts. 

CNV can be found in 2–18% of chronic CSC patients [141,142,143,144]. A recent report identified older age, wide PED width at diagnosis and recurrent episodes of CSC as independent risk factors for the development of CNV [145]. In addition, the authors found that CSC patients with secondary CNV had lower choriocapillary flow densities than those without secondary CNV. The presence of a PED at initial diagnosis was associated with an approximately 12-fold increased risk of secondary CNV. In addition, it has been suggested that DLS in eyes with CSC usually correspond to flat irregular PED and is associated with the development of CNV [146]. The neovascular activity of flat irregular PED at CSC initial phase could be considered of low grade. However, an active form of CNV may appear later, in the chronic phase. Recently, an OCT-A study of chronic CSC revealed that CNV was detected in one-third of eyes with flat irregular PED, which usually corresponded to DLS [134]. 

CNV tends to appear gradually, especially in patients over the age of 50 and/or with longstanding disease (Figure 4). Subretinal leakage from type 1 (sub-RPE) CNV due to pachychoroid neovasculopathy may resemble uncomplicated chronic CSC [114,143]. CNV can be identified using multimodal imaging techniques such as OCT, FA, ICGA, and—in particular—OCT-A. An example of OCT-A features in a CSC eye with sub-RPE neovascular network is provided in Figure 5. 

On FAF there is a mixed pattern of changes (both hypo- and hyperautofluorescent), with an irregular autofluorescence in the lower portion of the posterior pole (gravitational disposition of fluid). A hyperfluorescent lesion with perilesional leakage on FA (mainly hypocyanescent on ICGA) suggests the presence of an active CNV with a small SRF quantity. OCT-A clears up any doubt highlighting the sub-RPE neovascular network.

Therefore, it may happen that the initial diagnosis is CSC without CNV, despite the presence of a small CNV at that time. The clinician should suspect CNV in eyes with one or more of the following: “old age” at onset, a mid/hyperreflective signal under a flat irregular PED, a structure suggestive for CNV on OCT-A (Figure 5), and/or a well-demarcated CNV ‘plaque’ (with or without PCV component) on ICGA [147].

Because up to two-thirds of CSC patients with CNV can have a polypoidal component, ICG-A is a keytool for identifying and localizing these polypoidal structures [147]. De Salvo et al. [148] compared SD-OCT and ICGA levels of sensitivity and specificity in detecting idiopathic PCV and separating PCV from occult CNV. SD-OCT showed a 94.6% sensitivity and a 92.9% specificity.

The standard treatment for CSC complicated by active CNV is intravitreal anti-VEGF, possibly supplemented by HD- or HF-PDT. Several reports demonstrated good efficacy in these cases [149,150,151,152]. The MINERVA study found that intravitreal ranibizumab is effective in CNV with an unusual origin, including CNV due to CSC [151].

With respect to PCV, large randomized controlled trials based on EVEREST II and PLANET studies found that a combination of full-dose PDT and intravitreal ranibizumab or aflibercept may be beneficial [153,154]. In addition, it has been recently reported that 50% of PCV lesions were resolved after full-fluence PDT monotherapy, compared to 25% of lesions in patients who received anti-VEGF only [152]. As to the group of CSC patients with flat irregular PED in which thin neovascularization can be detected by OCT-A, the use of anti-VEGF therapy should be weighed carefully and deferred until active leakage becomes evident [135]. In addition, OCT-A, FA, and/or ICGA should be routinely performed to rule out the possibility of CNV presence in chronic CSC eyes with intraretinal fluid, as up to 45% of these cases may host CNV and should undergo the appropriate therapy [128].

OCT and OCT-A implemented significantly information provided by angiographies. SD-OCT is helpful in confirming the diagnosis of acute and chronic CSC and essential in determining the presence of features (SRF volume and height, SRF persistence, photoreceptors integrity, PED number, and subretinal fibrin) able to influence the natural course of the disease or the outcome of treatment procedures. Actually, some of these features are associated with a poor prognosis for final visual acuity. Moreover, nowadays, OCT-A is warmly recommended to identify CNV complicating chronic CSC, so avoiding the risk to miss the diagnosis and administer the wrong treatment.

## 3. Materials and Methods

We carried out an electronic search for relevant articles in PubMed from inception until December 2020. The workflow was arranged in compliance with the guidelines of the preferred reporting items for systematic reviews and meta-analyses (PRISMA) model. The main keywords used in combination included “central serous chorioretinopathy”, “imaging”, “fluorescein angiography”, “indocyanine green angiorgraphy”, “ structural optical coherence tomography”, “fundus autofluorescence”, and “optical coherence tomograhy angiography”.

At the end of December 2020 a total of 2245 articles were listed in Pubmed by using “central serous chorioretinopathy” as the primary search key. Subsequently, CSC was combined with the secondary search key and obtaining the following results respectively: “imaging” —714 articles—, “fluorescein angiography”—1129 articles—, “indocyanine green angiorgraphy” —374 articles—, “structural optical coherence tomography” —1171 articles—, “fundus autofluorescence”—999 articles—, “optical coherence tomograhy angiography (including acronyms OCTA and OCT-A)”—106 articles.

A first exclusion were review papers, case reports, articles not written in English, excluding from final analysis 281 review articles, 603 case reports, and 248 articles not written in English.

Two authors (S. D. P and P. I.) independently evaluated the preliminary results of the research using the title of the manuscript and the abstract as discriminating elements. In particular, the abstract was initially evaluated for the purpose of the studies. Manuscripts were considered if the primary purpose of the research included imaging studies and descriptions of morphological features of the retina in association with CSC with the previously mentioned imaging methods. Subsequently, attention was paid to studies describing imaging alterations or new anatomical findings in association with different therapeutical approaches. The list of manuscripts was further scrutinized for the methods section. In detail, we assessed the application of the imaging methodologies and the type of treatment administered. Further attention was focused on prospective studies and analysis of the most recent biomarkers. 

The final list produced by the two authors —composed of 213 articles— was then openly evaluated and discussed with the other authors, assessing the relevance of the studies with the aims of the current research; in particular, all the authors were aware that the current review was carried out with the aim of offering points of discussion and personal critical review. In detail, each other author received the complete list of the first selection who independently examined the title and abstract of the manuscript. The relevance of the articles to be taken into consideration was collectively discussed, and personal comments were exposed. A total of 128 manuscripts were considered relevant and evaluated in the current review. 

## 4. Conclusions

In conclusion, multimodal imaging technology in CSC developed very rapidly in the past few years and opened new perspectives about the etiology and physiopathology of this disease. Aside from eyes with a full recovery from their first episode of acute CSC, the identification of baseline biomarkers and indicators associated with a higher risk for permanent severe damage to RPE and photoreceptors was obtained combining information from FA, ICGA, FAF, OCT, and OCT-A. On this basis, patients that do not recover spontaneously or have multiple recurrent episodes saw their chance to avoid severe and untreatable visual loss significantly increase, considering that now they can receive the most appropriate treatment. Special attention must be paid to CSC eyes at high risk to develop CNV, since their chance to preserve vision is directly linked to a timely diagnosis and the administration of adequate therapy.

## References

[1] Klein M.L., Van Buskirk E.M., Friedman E., Gragoudas E., Chandra S. (1974). Experience with Nontreatment of Central Serous Choroidopathy. Arch. Ophthalmol..

[2] Yannuzzi L.A. (2010). Central Serous Chorioretinopathy: A Personal Perspective. Am. J. Ophthalmol..

[3] Breukink M.B., Dingemans A.J., Hollander A.I.D., Keunen J.E., MacLaren R.E., Fauser S., Querques G., Hoyng C.B., Downes S.M., Boon C.J. (2016). Chronic central serous chorioretinopathy: Long-term follow-up and vision-related quality of life. Clin. Ophthalmol..

[4] Mrejen S., Balaratnasingam C., Kaden T.R., Bottini A., Singh S.R., Bhavsar K.V., Yannuzzi N.A., Klufas M.A., Chen K.C., Yu S. (2019). Long-term Visual Outcomes and Causes of Vision Loss in Chronic Central Serous Chorioretinopathy. Ophthalmology.

[5] Nicholson B., Noble J., Forooghian F., Meyerle C. (2013). Central Serous Chorioretinopathy: Update on Pathophysiology and Treatment. Surv. Ophthalmol..

[6] Gass J.D.M. (1997). Stereoscopic Atlas of Macular Diseases: Diagnosis and Treatment.

[7] Desmettre T., Devoisselle J., Mordon S. (2000). Fluorescence Properties and Metabolic Features of Indocyanine Green (ICG) as Related to Angiography. Surv. Ophthalmol..

[8] Destro M., Puliafito C.A. (1989). Indocyanine Green Videoangiography of Choroidal Neovascularization. Ophthalmology.

[9] Hayashi K., Hasegawa Y., Tazawa Y., DeLaey J.J. (1989). Clinical application of indocyanine angiography to choroidal ne-ovascularization. Jpn. J. Ophthalmol..

[10] Yamada K., Hayasaka S., Setogawa T. (1992). Fluorescein-angiographic patterns in patients with central serous chori-oretinopathy at the initial visit. Ophthalmologica.

[11] Shahin M. (2013). Angiographic characteristics of central serous chorioretinopathy in an Egyptian population. Int. J. Ophthalmol..

[12] Bujarborua D., Nagpal P.N., Deka M. (2009). Smokestack leak in central serous chorioretinopathy. Graefe’s Arch. Clin. Exp. Ophthalmol..

[13] Yannuzzi L.A., Shakin J.L., Fisher Y.L., Altomonte M.A. (1984). Peripheral retinal detachments and retinal pigment epithe-lial atrophic tracts secondary to central serous pigment epitheliopathy. Ophthalmology.

[14] Gäckle H.C., Lang G.E., Freissler K.A., Lang G.K. (1998). Central serous chorioretinopathy. Clinical, fluorescein angiography and demographic aspects. Der Ophthalmol..

[15] Hayashi K., Hasegawa Y., Tokoro T. (1986). Indocyanine green angiography of central serous chorioretinopathy. Int. Ophthalmol..

[16] Wong R.L., Lai T.Y.Y. (2013). Polypoidal Choroidal Vasculopathy: An Update on Therapeutic Approaches. J. Ophthalmic Vis. Res..

[17] Cheung C.Y.-L., Laude A., Wong W., Mathur R., Chan C.M., Wong E., Wong D., Wong T.Y., Lim T.H. (2015). Improved specificity of polypoidal choroidal vasculopathy diagnosis using a modified everest criteria. Retina.

[18] Koh A.H., Chen L.J., Chen S.J., Chen Y., Giridhar A., Iida T., Kim H., Lai T.Y.Y., Lee W.K., Li X. (2013). Polypoidal choroidal vasculopathy: Evidence-based guide-lines for clinical diagnosis and treatment. Retina.

[19] Tan C.S., Ngo W.K., Chen J.P., Tan N.W., Lim T.H., EVEREST Study Group (2015). EVEREST study report 2: Imaging and grading protocol, and baseline characteristics of a randomised controlled trial of polypoidal choroidal vasculopa-thy. Br. J. Ophthalmol..

[20] Lim T.H., Laude A., Tan C.S. (2010). Polypoidal choroidal vasculopathy: An angiographic discussion. Eye.

[21] Tan C.S., Ngo W.K., Lim L.W., Tan N.W., Lim T.H., EVEREST Study Group (2016). EVEREST study report 3: Diagnostic chal-lenges of polypoidal choroidal vasculopathy: Lessons learnt from screening failures in the EVEREST study. Graefes Arch. Clin. Exp. Ophthalmol..

[22] Menchini U., Virgili G., Lanzetta P., Ferrari E. (1997). Indocyanine green angiography in central serous chorioretinopathy. Int. Ophthalmol..

[23] Shiraki K., Moriwaki M., Matsumoto M., Yanagihara N., Yasunari T., Miki T. (1997). Long-term follow-up of severe central serous chorioretinopathy using indocyanine green angiography. Int. Ophthalmol..

[24] Tsujikawa A., Ojima Y., Yamashiro K., Ooto S., Tamura H., Nakagawa S., Yoshimura N. (2010). Punctate hyperfluorescent spots as-sociated with central serous chorioretinopathy as seen on indocyanine green angiography. Retina.

[25] Li L., Li D.-H., Yang Z.-K., Bian A.-L., Chen Y.-X., Dong F. (2012). Analysis of fundus fluorescein angiography, indocyanine green angiography and choroidal thickness in central serous chorioretinopathy. Zhonghua Yan Ke Za Zhi Chin. J. Ophthalmol..

[26] Park S.J., Kim B.H., Park K.H., Woo S.J. (2014). Punctate hyperfluorescence spot as a common choroidopathy of central se-rous chorioretinopathy and polypoidal choroidal vasculopathy. Am. J. Ophthalmol..

[27] Pang C.E., Shah V.P., Sarraf D., Freund K.B. (2014). Ultra-Widefield Imaging with Autofluorescence and Indocyanine Green Angiography in Central Serous Chorioretinopathy. Am. J. Ophthalmol..

[28] Hirahara S., Yasukawa T., Kominami A., Nozaki M., Ogura Y. (2016). Densitometry of Choroidal Vessels in Eyes with and without Central Serous Chorioretinopathy by Wide-Field Indocyanine Green Angiography. Am. J. Ophthalmol..

[29] Robertson D.M., Ilstrup D. (1983). Direct, Indirect, and Sham Laser Photocoagulation in the Management of Central Serous Chorioretinopathy. Am. J. Ophthalmol..

[30] Robertson D.M. (1986). Argon Laser Photocoagulation Treatment in Central Serous Chorioretinopathy. Ophthalmology.

[31] Ficker L., Vafidis G., While A., Leaver P. (1988). Long-term follow-up of a prospective trial of argon laser photocoagula-tion in the treatment of central serous retinopathy. Br. J. Ophthalmol..

[32] Chen S.-N., Hwang J.-F., Tseng L.-F., Lin C.-J. (2008). Subthreshold Diode Micropulse Photocoagulation for the Treatment of Chronic Central Serous Chorioretinopathy with Juxtafoveal Leakage. Ophthalmology.

[33] Luttrull J.K. (2016). Low-intensity/high-density subthreshold diode micropulse laser for central serous chorioretinopa-thy. Retina.

[34] Malik K.J., Sampat K.M., Mansouri A., Steiner J.N., Glaser B.M. (2015). Low-intensity/high-density subthreshold micropulse diode laser for chronic central serous chorioretinopathy. Retina.

[35] van Dijk E.H.C., Fauser S., Breukink M.B., Blanco-Garavito R., Groenewoud J.M.M., Keunen J.E.E., Peters P.J.H., Dijkman G., Souied E.H., MacLaren R.E. (2018). Half-dose photodynamic therapy ver-sus high-density subthreshold micropulse laser treatment in patients with chronic central serous chorioretinopathy: The Place trial. Ophthalmology.

[36] Chhablani J., Rani P.K., Mathai A., Jalali S., Kozak I. (2014). Navigated focal laser photocoagulation for central serous chorioretinopathy. Clin. Ophthalmol..

[37] Müller B., Tatsios J., Klonner J., Pilger D., Joussen A.M. (2018). Navigated laser photocoagulation in patients with non-resolving and chronic central serous chorioretinopathy. Graefe’s Arch. Clin. Exp. Ophthalmol..

[38] van Rijssen T.J., van Dijk E.H.C., Scholz P., Breukink M.B., Blanco-Garavito R., Souied E.H., Keunen J.E.E., MacLaren R.E., Querques G., Fauser S. (2019). Focal and diffuse chronic central serous chorioretinopathy treated with half-dose photodynamic therapy or subthreshold micropulse laser. Am. J. Ophthalmol..

[39] Dhirani N.A., Yang Y., Somani S. (2017). Long-term outcomes in half-dose verteporfin photodynamic therapy for chronic central serous retinopathy. Clin. Ophthalmol..

[40] Haga F., Maruko R., Sato C., Kataoka K., Ito Y., Terasaki H. (2017). Long-term prognostic factors of chronic central serous chorioretinopathy after half-dose photodynamic therapy: A 3-year follow-up study. PLoS ONE.

[41] Fujita K., Imamura Y., Shinoda K., Matsumoto C.S., Mizutani Y., Hashizume K., Mizota A., Yuzawa M. (2015). One-Year Outcomes with Half-Dose Verteporfin Photodynamic Therapy for Chronic Central Serous Chorioretinopathy. Ophthalmology.

[42] Lai F.H., Ng D.S., Bakthavatsalam M., Chan V.C., Young A.L., Luk F.O., Tsang C.W., Brelén M.E. (2016). A Multicenter Study on the Long-term Outcomes of Half-dose Photodynamic Therapy in Chronic Central Serous Chorioretinopathy. Am. J. Ophthalmol..

[43] Hayashida M., Miki A., Honda S., Nakamura M. (2020). Comparison between the outcomes of fluorescein angiography-guided and indocyanine green angiography-guided half-time photodynamic therapy for central serous chorioretinopathy. Photodiagnosis Photodyn. Ther..

[44] Kim K.S., Lee W.K., Lee S.B. (2014). Half-dose photodynamic therapy targeting the leakage point on the fluorescein angi-ography in acute central serous chorioretinopathy: A pilot study. Am. J. Ophthalmol..

[45] Ozkaya A., Alkin Z., Ozveren M., Yazici A.T., Taskapili M. (2016). The time of resolution and the rate of recurrence in acute central serous chorioretinopathy following spontaneous resolution and low-fluence photodynamic therapy: A case–control study. Eye.

[46] Mohabati D., Boon C.J.F., Yzer S. (2020). Risk of Recurrence and Transition to Chronic Disease in Acute Central Serous Chorioretinopathy. Clin. Ophthalmol..

[47] Inoue R., Sawa M., Tsujikawa M., Gomi F. (2010). Association between the efficacy of photodynamic therapy and indo-cyanine green angiography findings for central serous chorioretinopathy. Am. J. Ophthalmol..

[48] van Rijssen T.J., van Dijk E.H.C., Dijkman G., Boon C.J.F. (2018). Clinical characteristics of chronic central serous chorioreti-nopathy patients with insufficient response to reduced-settings photodynamic therapy. Graefes Arch. Clin. Exp. Ophthalmol..

[49] Mohabati D., Van Dijk E.H., Van Rijssen T.J., De Jong E.K., Breukink M.B., Martinez-Ciriano J.P., Dijkman G., Hoyng C.B., Fauser S., Yzer S. (2018). Clinical spectrum of severe chronic central serous chorioretinopathy and outcome of photodynamic therapy. Clin. Ophthalmol..

[50] Holz F.G., Schmitz-Valckenberg S., Spaide R.F., Bird A.C. (2007). Atlas of Fundus Autofluorescence Imaging.

[51] Delori F.C., Dorey C.K., Staurenghi G., Arend O., Goger D.G., Weiter J.J. (1995). In vivo fluorescence of the ocular fundus ex-hibits retinal pigment epithelium lipofuscin characteristics. Investig. Ophthalmol. Vis. Sci..

[52] Brunk U., Wihlmark U., Wrigstad A., Roberg K., Nilsson S.-E. (1995). Accumulation of lipofuscin within retinal pigment epithelial cells results in enhanced sensitivity to photo-oxidation. Gerontology.

[53] Kim S.K., Kim S.W., Oh J., Huh K. (2013). Near-infrared and short-wavelength autofluorescence in resolved central serous chorioretinopathy: Association with outer retinal layer abnormalities. Am. J. Ophthalmol..

[54] Zhang P., Wang H.Y., Zhang Z.F., Sun D.J., Zhu J.T., Li J., Wang J.S. (2015). Fundus autofluorescence in central serous chori-oretinopathy: Association with spectral-domain optical coherence tomography and fluorescein angiography. Int. J. Ophthalmol..

[55] Pryds A., Larsen M. (2013). Foveal function and thickness after verteporfin photodynamic therapy in central serous chorioretinopathy with hyperautofluorescent subretinal deposits. Retina.

[56] Spaide R.F., Klancnik J.M., Klancnik J. (2005). Fundus autofluorescence and central serous chorioretinopathy. Ophthalmology.

[57] Lee W.J., Lee J.H., Lee B.R. (2016). Fundus autofluorescence imaging patterns in central serous chorioretinopathy according to chronicity. Eye.

[58] Framme C., Walter A., Gabler B., Roider J., Sachs H.G., Gabel V.-P. (2005). Fundus autofluorescence in acute and chronic-recurrent central serous chorioretinopathy. Acta Ophthalmol. Scand..

[59] Dinc U.A., Tatlipinar S., Yenerel M., Görgün E., Ciftci F. (2011). Fundus autofluorescence in acute and chronic central se-rous chorioretinopathy. Clin. Exp. Optom..

[60] Ayata A., Tatlipinar S., Kar T., Unal M., Ersanli D., Bilge A.H. (2009). Near-infrared and short-wavelength autofluo-rescence imaging in central serous chorioretinopathy. Br. J. Ophthalmol..

[61] Matsumoto H., Kishi S., Sato T., Mukai R. (2011). Fundus Autofluorescence of Elongated Photoreceptor Outer Segments in Central Serous Chorioretinopathy. Am. J. Ophthalmol..

[62] Zola M., Chatziralli I., Menon D., Schwartz R., Hykin P., Sivaprasad S. (2018). Evolution of fundus autofluorescence patterns over time in patients with chronic central serous chorioretinopathy. Acta Ophthalmol..

[63] Han J., Cho N.S., Kim K., Kim E.S., Kim D.G., Kim J.M., Yu S. (2020). Fundus autofluorescence patterns in central serous chorioretinopathy. Retina.

[64] Huang D., Swanson E.A., Lin C.P., Schuman J.S., Stinson W.G., Chang W., Hee M.R., Flotte T., Gregory K., Puliafito C.A. (1991). Optical coherence tomography. Science.

[65] Wojtkowski M., Bajraszewski T., Gorczyńska I., Targowski P., Kowalczyk A., Wasilewski W., Radzewicz C. (2004). Ophthalmic imaging by spectral optical coherence tomography. Am. J. Ophthalmol..

[66] Spaide R.F., Koizumi H., Pozonni M.C. (2008). Enhanced Depth Imaging Spectral-Domain Optical Coherence Tomography. Am. J. Ophthalmol..

[67] Adhi M., Liu J.J., Qavi A.H., Grulkowski I., Lu C.D., Mohler K.J., Ferrara D., Kraus M.F., Baumal C., Witkin A.J. (2014). Choroidal Analysis in Healthy Eyes Using Swept-Source Optical Coherence Tomography Compared to Spectral Domain Optical Coherence Tomography. Am. J. Ophthalmol..

[68] Kim D.Y., Fingler J., Zawadzki R.J., Park S.S., Morse L.S., Schwartz D.M., Fraser S., Werner J.S. (2013). Optical imaging of the chorioretinal vasculature in the living human eye. Proc. Natl. Acad. Sci. USA.

[69] Choi W., Mohler K.J., Potsaid B., Lu C.D., Liu J.J., Jayaraman V., Cable A.E., Duker J.S., Huber R., Fujimoto J.G. (2013). Choriocapillaris and choroidal microvasculature imaging with ultrahigh speed OCT angiography. PLoS ONE.

[70] Schwartz D.M., Fingler J., Kim D.J., Zawadzki R.J., Motrse L.S., Park S.S., Fraser S.E., Werner J.S. (2014). Phase-variance optical coherence tomography: A technique for noninvasive angiography. Ophthalmology.

[71] Kozak I., Payne J.F., Schatz P., Al-Kahtani E., Winkler M. (2017). Teleophthalmology image-based navigated retinal laser therapy for diabetic macular edema: A concept of retinal telephotocoagulation. Graefe’s Arch. Clin. Exp. Ophthalmol..

[72] Ganekal S., Nair U.K., Soman M., Nair K. (2012). Correlation of spectral domain optical coherence tomography findings in acute central serous chorioretinopathy with visual acuity. Clin. Ophthalmol..

[73] Maltsev D.S., Kulikov A.N., Chhablani J. (2018). Topographic-guided identification of leakage point in central serous cho-rioretinopathy: A base for fluorescein-angiography free focal laser photocoagulation. Br. J. Ophthalmol..

[74] Maltsev D.S., Kulikov A.N., Chhablani J. (2019). Clinical Application of Fluorescein Angiography-Free Navigated Focal Laser Photocoagulation in Central Serous Chorioretinopathy. Ophthalmic Surg. Lasers Imaging Retin..

[75] Rajesh B., Kaur A., Giridhar A., Gopalakrishnan M. (2017). “Vacuole” sign adjacent to retinal pigment epithelial defects on spectral domain optical coherence tomography in central serous chorioretinopathy associated with subretinal fibrin. Retina.

[76] Hata M., Oishi A., Shimozono M., Mandai M., Nishida A., Kurimoto Y. (2013). Early changes in foveal thickness in eyes with central serous chorioretinopathy. Retina.

[77] Wang M., Sander B., Lund-Andersen H., Larsen M. (1999). Detection of shallow detachments in central serous chorioretinopathy. Acta Ophthalmol. Scand..

[78] Wang M.S., Sander B., Larsen M. (2002). Retinal atrophy in idiopathic central serous chorioretinopathy11InternetAdvance publication at ajo.com Feb 28, 2002. Am. J. Ophthalmol..

[79] Wang M., Sander B., la Cour M., Larsen M. (2005). Clinical characteristics of subretinal deposits in central serous chorioretinopathy. Acta Ophthalmol. Scand..

[80] Iacono P., Battaglia Parodi M., Papayannis A., La Spina C., Varano M., Bandello F. (2015). Acute central serous chorioreti-nopathy: A correlation study between fundus autofluorescence and spectral-domain OCT. Graefes Arch. Clin. Exp. Ophthalmol..

[81] Koss M.J., Beger I., Koch F.H. (2011). Subthreshold diode laser micropulse photocoagulation versus intravitreal injections of bevacizumab in the treatment of central serous chorioretinopathy. Eye.

[82] Kretz F.T., Beger I., Koch F., Nowomiejska K., Auffarth G.U., Koss M.J. (2015). Randomized clinical trial to compare micro-pulse photocoagulation versus half-dose verteporfin photodynamic therapy in the treatment of central serous chorioretinopathy. Ophthalmic Surg. Lasers Imaging Retina.

[83] Park Y.G., Kang S., Kim M., Yoo N., Roh Y.J. (2017). Selective retina therapy with automatic real-time feedback-controlled dosimetry for chronic central serous chorioretinopathy in Korean patients. Graefe’s Arch. Clin. Exp. Ophthalmol..

[84] Arsan A., Kanar H.S., Sonmez A. (2018). Visual outcomes and anatomic changes after sub-threshold micropulse yellow laser (577-nm) treatment for chronic central serous chorioretinopathy: Long-term follow-up. Eye.

[85] Scholz P., Altay L., Fauser S. (2016). Comparison of subthreshold micropulse laser (577 nm) treatment and half-dose photodynamic therapy in patients with chronic central serous chorioretinopathy. Eye.

[86] Scholz P., Altay L., Fauser S. (2017). A Review of Subthreshold Micropulse Laser for Treatment of Macular Disorders. Adv. Ther..

[87] Maruko I., Iida T., Sugano Y., Ojima A., Ogasawara M., Spaide R.F. (2010). Subfoveal Choroidal Thickness after Treatment of Central Serous Chorioretinopathy. Ophthalmology.

[88] van Dijk E.H.C., Dijkman G., Theelen T., Hoyng C.B., Boon C.J.F. (2018). Short-term findings on optical coherence tomography and microperimetry in chronic central serous chorioretinopathy patients treated with half-dose photodynamic therapy. Retin Cases Brief Rep..

[89] Maruko I., Iida T., Sugano Y., Furuta M., Sekiryu T. (2011). One-year choroidal thickness results after photodynamic therapy for central serous chorioretinopathy. Retina.

[90] Cardillo Piccolino F., De La Longrais R.R., Manea M., Cicinelli S. (2008). Posterior cystoid retinal degeneration in central serous chorioretinopathy. Retina.

[91] Chung C.Y., Chan Y.Y., Li K.K.W. (2018). Angiographic and tomographic prognostic factors of chronic central serous chorioretinopathy treated with half-dose photodynamic therapy. Ophthalmologica.

[92] Nicolo M., Zoli D., Musolino M., Traverso C.E. (2012). Association between the efficacy of half-dose photodynamic therapy with indocyanine green angiography and optical coherence tomography findings in the treatment of central serous chorioretinopathy. Am. J. Ophthalmol..

[93] Wu J.-S., Chen S.-N. (2019). Optical Coherence Tomography Angiography for Diagnosis of Choroidal Neovascularization in Chronic Central Serous Chorioretinopathy after Photodynamic Therapy. Sci. Rep..

[94] Herold T.R., Prause K., Wolf A., Mayer W.J., Ulbig M.W. (2014). Spironolactone in the treatment of central serous chori-oretinopathy—A case series. Graefes Arch. Clin. Exp. Ophthalmol..

[95] Bousquet E., Beydoun T., Rothschild P.R., Bergin C., Zhao M., Batista R., Brandely M.L., Couraud B., Farman N., Gaudric A. (2015). Spironolactone for nonresolving central serous chorioretinopathy: A random-ized controlled crossover study. Retina.

[96] Chin E.K., Almeida D.R., Roybal C.N., Niles P.I., Gehrs K.M., Sohn E.H., Boldt H.C., Russell S.R., Folk J.C. (2015). Oral mineralo-corticoid antagonists for recalcitrant central serous chorioretinopathy. Clin. Ophthalmol..

[97] Chai Y., Liu R.-Q., Yi J.-L., Ye L.-H., Zou J., Jiang N., Shao Y. (2016). Clinical research of fenofibrate and spironolactone for acute central serous chorioretinopathy. Int. J. Ophthalmol..

[98] Daruich A., Matet A., Dirani A., Gallice M., Nicholson L., Sivaprasad S., Behar-Cohen F. (2016). Oral mineralocorti-coid-receptor antagonists: Real-life experience in clinical subtypes of nonresolving central serous chorioretinopathy with chronic epitheliopathy. Transl. Vis. Sci. Technol..

[99] Kapoor K.G., Wagner A.L. (2016). Mineralocorticoid Antagonists in the Treatment of Central Serous Chorioretinopathy: A Comparative Analysis. Ophthalmic Res..

[100] Aghdam K.A., Falavarjani K.G., Amirsardari A., Habibi A., Eshaghi A., Bakhti S. (2017). Visual and anatomical outcomes of spironolactone therapy in patients with chronic central serous chorioretinopathy. J. Ophthalmic Vis. Res..

[101] Herold T.R., Rist K., Priglinger S.G., Ulbig M.W., Wolf A. (2017). Long-term results and recurrence rates after spironolac-tone treatment in non-resolving central serous chorio-retinopathy (cscr). Graefes Arch. Clin. Exp. Ophthalmol..

[102] Pichi F., Carrai P., Ciardella A., Behar-Cohen F., Nucci P. (2017). Central Serous Chorioretinopathy Study Group. Comparison of two mineralcorticosteroids receptor antagonists for the treatment of central serous chorioretinopathy. Int. Ophthalmol..

[103] Sacconi R., Baldin G., Carnevali A., Querques L., Rabiolo A., Marchini G., Bandello F. (2018). Response of central serous chorioretinopathy evaluated by multimodal retinal imaging. Eye.

[104] Bousquet E., Dhundass M., Lejoyeux R., Shinojima A., Krivosic V., Mrejen S., Gaudric A., Tadayoni R. (2019). Predictive Factors of Response to Mineralocorticoid Receptor Antagonists in Nonresolving Central Serous Chorioretinopathy. Am. J. Ophthalmol..

[105] Cakir B., Fischer F., Ehlken C., Buhler A., Stahl A., Schlunck G., Bohringer D., Agostini H., Lange C. (2016). Clinical experi-ence with eplerenone to treat chronic central serous chorioretinopathy. Graefes Arch. Clin. Exp. Ophthalmol..

[106] Borrelli E., Zuccaro B., Zucchiatti I., Parravano M., Querques L., Costanzo E., Sacconi R., Prascina F., Scarinci F., Bandello F. (2019). Optical coherence tomography parameters as predictors of treatment response to ep-lerenone in central serous chorioretinopathy. J. Clin. Med..

[107] Willcox A., Culliford L., Ellis L., Rogers C.A., Cree A., Chakravarthy U., Ennis S., Behar-Cohen F., Reeves B.C., Si-Vaprasad S. (2019). Clinical efficay of eplerenone versus placebo for central serous chorioretinopathy: Study pro-tocol for the VICI randomised controlled trial. Eye.

[108] Lotery A., Sivaprasad S., O’Connell A., Harris R.A., Culliford L., Ellis L., Cree A., Madhusudhan S., Behar-Cohen F., Chakravarthy U. (2020). Eplerenone for chronic central serous cho-rioretinopathy in patients with active, previously untreated disease for more than 4 months (VICI): A randomised, double-blind, placebo-controlled trial. Lancet.

[109] Yang L., Jonas J.B., Wei W. (2013). Optical Coherence Tomography–Assisted Enhanced Depth Imaging of Central Serous Chorioretinopathy. Investig. Opthalmol. Vis. Sci..

[110] Dansingani K.K., Balaratnasingam C., Naysan J., Freund K.B. (2016). En face imaging of pachychoroid spectrum disorders with swept-source optical coherence tomography. Retina.

[111] Shinojima A., Fujita K., Mori R., Kawamura A., Yuzawa M., Yasukawa T. (2016). Investigation of the Etiology of Central Serous Chorioretinopathy Using En-Face Optical Coherence Tomography and Indocyanine Green Angiography. Ophthalmologica.

[112] Lee W.J., Lee J.W., Park S.H., Lee B.R. (2017). En face choroidal vascular feature imaging in acute and chronic central se-rous chorioretinopathy using swept source optical coherence tomography. Br. J. Ophthalmol..

[113] Warrow D.J., Hoang Q.V., Freund K.B. (2013). Pachychoroid pigment epitheliopathy. Retina.

[114] Pang C.E., Freund K.B. (2015). Pachychoroid neovasculopathy. Retina.

[115] Dirani A., Matet A., Daruich A.B., Parvin P., Elalouf M., Ambresin A., Mantel I., Behar-Cohen F.F. (2015). Risk factors and choroidal changes in patients with unilateral versus bilateral chronic active CSCR. Investig. Ophthalmol. Vis. Sci..

[116] Hanumunthadu D., Matet A., Rasheed M.A., Goud A., Vuppurabina K.K., Chhablani J. (2019). Evaluation of choroidal hyperreflective dots in acute and chronic central serous chorioretinopathy. Indian J. Ophthalmol..

[117] Lee H., Lee J., Chung H., Kim H.C. (2016). Baseline spectral domain optical coherence tomographic hyperreflective foci as a predictor of visual outcome and recurrence for central serous chorioretinopathy. Retina.

[118] Song I.S., Shin Y.U., Lee B.R. (2012). Time-periodic characteristics in the morphology of idiopathic central serous chorioretinopathy evaluated by volume scan using spectral-domain optical coherence tomography. Am. J. Ophthalmol..

[119] Mohabati D., van Rijssen T.J., van Dijk E.H., Luyten G.P., Missotten T.O., Hoyng C.B., Boon C.J. (2018). Clinical characteristics and long-term visual outcome of severe phenotypes of chronic central serous chorioretinopathy. Clin. Ophthalmol..

[120] Otsuka S., Ohba N., Nakao K. (2002). A Long-term follow-up study of severe variant of central serous chorioretinopathy. Retina.

[121] Castro-Correia J., Coutinho M.F., Rosas V., Maia J. (1992). Long-term follow-up of central serous retinopathy in 150 patients. Doc. Ophthalmol..

[122] Iida T., Yannuzzi L.A., Spaide R.F., Borodoker N., Carvalho C.A., Negrao S. (2003). Cystoid macular degeneration in chronic central serous chorioretinopathy. Retina.

[123] Staurenghi G., Lai T.Y.Y., Mitchell P., Wolf S., Wenzel A., Li J., PROMETHEUS Study Group (2018). Efficacy and safety of ranibizumab 0.5 mg for the treatment of macular edema resulting from uncommon causes: Twelve-month findings from prometheus. Ophthalmology.

[124] Piccolino F.C., De La Longrais R.R., Manea M., Cicinelli S., Ravera G. (2008). Risk factors for posterior cystoid retinal de-generation in central serous chorioretinopathy. Retina.

[125] Mohabati D., Hoyng C.B., Yzer S., Boon C.J. (2019). Clinical characteristics and outcome of posterior cystoid macular degeneration in chronic central serous chorioretinopathy. Retina.

[126] Sahoo N.K., Mishra S.B., Iovino C., Singh S.R., Munk M.R., Berger L., Peiretti E., Chhablani J. (2019). Optical coherence to-mography angiography findings in cystoid macular degeneration associated with central serous chorioretinopathy. Br. J. Ophthalmol..

[127] Daruich A., Matet A., Dirani A., Bousquet E., Zhao M., Farman N., Jaisser F., Behar-Cohen F. (2015). Central serous chori-oretinopathy: Recent findings and new physiopathology hypothesis. Prog. Retin. Eye Res..

[128] Ahlers C., Geitzenauer W., Stock G., Golbaz I., Schmidt W.M., Prünte C. (2009). Alterations of intraretinal layers in acute central serous chorioretinopathy. Acta Ophthalmol..

[129] Hirami Y., Tsujikawa A., Sasahara M., Gotoh N., Tamura H., Otani A., Mandai M., Yoshimura N. (2007). Alterations of retinal pigment epithelium in central serous chorioretinopathy. Clin. Exp. Ophthalmol..

[130] Sheth J., Anantharaman G., Chandra S., Sivaprasad S. (2018). “Double-layer sign” on spectral domain optical coherence tomography in pachychoroid spectrum disease. Indian J. Ophthalmol..

[131] Hage R., Mrejen S., Krivosic V., Quentel G., Tadayoni R., Gaudric A. (2015). Flat irregular retinal pigment epithelium de-tachments in chronic central serous chorioretinopathy and choroidal neovascularization. Am. J. Ophthalmol..

[132] Dansingani K.K., Balaratnasingam C., Klufas M.A., Sarraf D., Freund K.B. (2015). Optical coherence tomography angi-ography of shallow irregular pigment epithelial detachments in pachychoroid spectrum disease. Am. J. Ophthalmol..

[133] Sato T., Kishi S., Watanabe G., Matsumoto H., Mukai R. (2007). Tomographic features of branching vascular networks in polypoidal vhoroidal vasculopathy. Retina.

[134] Bousquet E., Bonnin S., Mrejen S., Krivosic V., Tadayoni R., Gaudric A. (2018). Optical coherence tomography angiography of flat irregular pigment epithelium detachment in chronic central serous chorioretinopathy. Retina.

[135] Quaranta-El Maftouhi M., El Maftouhi A., Eandi C.M. (2015). Chronic central serous chorioretinopathy imaged by optical coherence tomographic angiography. Am. J. Ophthalmol..

[136] Bonini Filho M.A., de Carlo T.E., Ferrara D., Adhi M., Baumal C.R., Witkin A.J., Waheed N.K. (2015). Association of choroidal neovascularization and central serous chorioretinopathy with optical coherence tomography angiography. JAMA Ophthalmol..

[137] de Carlo T.E., Rosenblatt A., Goldstein M., Baumal C.R., Loewenstein A., Duker J.S. (2016). Vascularization of irregular retinal pigment epithelial detach-ments in chronic central serous chorioretinopathy evaluated with OCT angiography. Ophthalmic Surg. Lasers Imaging Retina.

[138] Yu J., Jiang C., Xu G. (2014). Study of subretinal exudation and consequent changes in acute central serous chorioreti-nopathy by optical coherence tomography. Am. J. Ophthalmol..

[139] Balaratnasingam C., Bailey Freund K., Tan A.M., Mrejen S., Hunyor A.P., Keegan D.J., Dansingani K.K., Dayani P.N., Barbazetto I.A., Sarraf D. (2016). Bullous variant of central serous chorioretinopathy: Expansion of phenotypic features using multimodal imaging. Ophthalomolgy.

[140] Liang Z., Qu J., Huang L., Linghu D., Hu J., Jin E., Xu H., Li H., Tao Y., Xu X. (2020). Comparison of the outcomes of photodynamic therapy for central serous chorioretinopathy with or without subfoveal fibrin. Eye.

[141] Spaide R.F., Campeas L., Haas A., Yannuzzi L.A., Fisher Y.L., Guyer D.R., Slakter J.S., Sorenson J.A., Orlock D.A. (1996). Central Serous Chorioretinopathy in Younger and Older Adults. Ophthalmology.

[142] Loo R.H., Scott I.U., Flynn H.W., Gass J.D., Murray T.G., Lewis M.L., Rosenfeld P.J., Smiddy W.E. (2002). Factors associated with reduced visual acuity during longterm follow-up of patients with idiopathic central serous chorioretinopathy. Retina.

[143] Fung A.T., Yannuzzi L.A., Freund K.B. (2012). Type 1 (sub-retinal pigment epithelial) neovascularization in central serous chorioretinopathy masquerading as neovascular age-related macular degeneration. Retina.

[144] Nicholson B.P., Idris A.M.A., Bakri S.J. (2018). Central Serous Chorioretinopathy: Clinical Characteristics Associated with Visual Outcomes. Semin. Ophthalmol..

[145] Yeo J.H., Oh R., Kim Y.J., Kim J., Yoon J.H., Lee J.Y. (2020). Choroidal neovascularization secondary to central serous chori-oretinopathy: OCT angiography findings and risk factors. J. Ophthalmol..

[146] Lee G.I., Kim A.Y., Kang S.W., Cho S.C., Park K.H., Kim S.J., Kim K.T. (2019). Risk factors and outcomes of choroidal neovascularization secondary to central serous chorioretinopathy. Sci. Rep..

[147] van Rijssen T.J., van Dijk E.H.C., Yzerb S., Ohno-Matsuic K., Keunend J.E.E., Schlingemanne R.O., Sivaprasad S., Querques G., Downesi S.M., Fauserj S. (2019). Central serous chorioretinopathy: Towards an evi-dence-based treatment guideline. Prog. Retin. Eye Res..

[148] De Salvo G., Vaz-Pereira S., Keane P.A., Tufail A., Liew G. (2014). Sensitivity and specificity of spectral-domain optical coherence tomography in detecting idiopathic polypoidal choroidal vasculopathy. Am. J. Ophthalmol..

[149] Chhablani J., Kozak I., Pichi F., Chenworth M., Berrocal M.H., Bedi R., Singh R.P., Wu L., Meyerle C., Casella A.M. (2015). Outcomes of treatment of choroidal neovasculari-zation associated with central serous chorioretinopathy with intravitreal antiangiogenic agents. Retina.

[150] Chhablani J., Pichi F., Silva R., Casella A.M., Murthy H., Banker A., Nowilaty S.R., Carrai P., Nucci P., Arevalo J.F. (2016). Antiangiogenics in choroidal neovascularization associated with laser in central serous chorioretinopathy. Retina.

[151] Lai T.Y.Y., Staurenghi G., Lanzetta P., Holz F.G., Liew S.H.M., Desset-Brethes S., Staines H., Hykin P.G. (2018). Efficacy and safety of ranibizumab for the treatment of choroidal neovascularization due to uncommon cause: Twelve-month results of the minerva study. Retina.

[152] Peiretti E., Caminiti G., Serra R., Querques L., Pertile R., Querques G. (2018). Anti-vascular endothelial growth factor therapy versus photodynamic therapy in the treatment of choroidal neovascularization secondary to central serous chorioretinopathy. Retina.

[153] Koh A., Lai T.Y.Y., Takahashi K., Wong T.Y., Chen L.J., Ruamviboonsuk P., Tan C.S., Feller C., Margaron P., Lim T.H. (2017). Efficacy and safety of ranibizumab with or without verteporfin photodynamic therapy for polypoidal cho-roidal vasculopathy: A randomized clinical trial. JAMA Ophthalmol..

[154] Lee W.K., Iida T., Ogura Y., Chen S.J., Wong T.Y., Mitchell P., Cheung G.C.M., Zhang Z., Leal S., Ishibashi T. (2018). Efficacy and safety of intravitreal aflibercept for polypoidal choroidal vasculopathy in the planet study: A randomized clinical trial. JAMA Ophthalmol..

